# Plasmonic gadolinium oxide nanomatryoshkas: bifunctional magnetic resonance imaging enhancers for photothermal cancer therapy

**DOI:** 10.1093/pnasnexus/pgac140

**Published:** 2022-07-29

**Authors:** Luke Henderson, Oara Neumann, Yara Kadria-Vili, Burak Gerislioglu, James Bankson, Peter Nordlander, Naomi J Halas

**Affiliations:** Department of Chemistry, Rice University, 6100 Main St, Houston, TX 77005, USA; Laboratory for Nanophotonics, Rice University, 6100 Main St, Houston, TX 77005, USA; Laboratory for Nanophotonics, Rice University, 6100 Main St, Houston, TX 77005, USA; Department of Electrical and Computer Engineering, Applied Physics Program, Laboratory for Nanophotonics, Rice University, 6100 Main St, Houston, TX 77005, USA; Department of Chemistry, Rice University, 6100 Main St, Houston, TX 77005, USA; Laboratory for Nanophotonics, Rice University, 6100 Main St, Houston, TX 77005, USA; Department of Imaging Physics, The University of Texas MD Anderson Cancer Center, 1515 Holcombe Boulevard, TX 77030, USA; Laboratory for Nanophotonics, Rice University, 6100 Main St, Houston, TX 77005, USA; Department of Physics and Astronomy, Laboratory for Nanophotonics, Rice University, 6100 Main St, Houston, TX 77005, USA; Department of Imaging Physics, The University of Texas MD Anderson Cancer Center, 1515 Holcombe Boulevard, TX 77030, USA; Laboratory for Nanophotonics, Rice University, 6100 Main St, Houston, TX 77005, USA; Department of Electrical and Computer Engineering, Applied Physics Program, Laboratory for Nanophotonics, Rice University, 6100 Main St, Houston, TX 77005, USA; Department of Physics and Astronomy, Laboratory for Nanophotonics, Rice University, 6100 Main St, Houston, TX 77005, USA; Department of Chemistry, Rice University, 6100 Main St, Houston, TX 77005, USA; Laboratory for Nanophotonics, Rice University, 6100 Main St, Houston, TX 77005, USA; Department of Electrical and Computer Engineering, Applied Physics Program, Laboratory for Nanophotonics, Rice University, 6100 Main St, Houston, TX 77005, USA; Department of Physics and Astronomy, Laboratory for Nanophotonics, Rice University, 6100 Main St, Houston, TX 77005, USA

**Keywords:** nanomatryoshka, MRI contrast agents, gadolinium, gadolinium oxide, photothermal cancer therapy

## Abstract

Nanoparticle-assisted laser-induced photothermal therapy (PTT) is a promising method for cancer treatment; yet, visualization of nanoparticle uptake and photothermal response remain a critical challenge. Here, we report a magnetic resonance imaging-active nanomatryoshka (Gd_2_O_3_-NM), a multilayered (Au core/Gd_2_O_3_ shell/Au shell) sub-100 nm nanoparticle capable of combining T_1_ MRI contrast with PTT. This bifunctional nanoparticle demonstrates an r_1_ of 1.28 × 10^8^ mM^–1^ s^–1^, an MRI contrast enhancement per nanoparticle sufficient for T_1_ imaging in addition to tumor ablation. Gd_2_O_3_-NM also shows excellent stability in an acidic environment, retaining 99% of the internal Gd(3). This report details the synthesis and characterization of a promising system for combined theranostic nanoparticle tracking and PTT.

Significance Statement Photothermal cancer therapy using near-IR-absorbing nanoparticles is successfully transitioning into clinical practice for the treatment of early-stage prostate cancer, a highly noninvasive approach that virtually eliminates the deleterious side effects associated with conventional prostate cancer therapies. A major challenge in the optimization of this treatment protocol is to obtain information regarding the concentration of nanoparticles that have been taken up by tumors within the prostate. Accurate information regarding nanoparticle concentration in vivo would allow for the streamlining and optimization of photothermal ablation treatments, saving time and expense. Developing nanoparticle synthetic strategies that render photothermally active gold nanoparticles to be also MRI-active would likely provide a solution to this knowledge gap.

## Introduction

Near-infrared (NIR) plasmonic nanomaterials are a highly promising class of therapeutics for drug delivery and localized photothermal cancer therapy (PTT) (1–4). They can target tumors by exploiting tumor biology through the enhanced permeability and retention effect (5), and ablate tumor cells through localized PTT (6). In addition, chemotherapeutic drugs or photosensitizers can be loaded on the nanoparticle (NP) and released upon near-infrared photothermal stimulus, further improving treatment outcomes compared to PTT alone (1, 7–9).

We have previously reported the synthesis and application of metallodielectric layered nanostructures we called nanomatryoshkas (NMs), composed of an Au core, an interstitial silica layer, and an outer Au shell (10, 11). NMs are an excellent vehicle for the passive targeting and treatment of solid tumors due their compact size (∼100 nm), biocompatibility, long circulation time, and efficient NIR light-to-heat conversion (11, 12). Similar to Au nanoshells, the outer Au shell can provide a scaffold for additional functionalization such as active targeting (13) and controlled drug release (14) to expand clinical applications and improve therapy outcomes.

To optimize these therapies in clinical applications, there is a crucial need to be able to monitor and measure NP accumulation in the body before, during, and after treatment. Imaging agents, such as fluorophores, radioisotopes, or MRI-contrast agents, can be added to therapeutic NPs to visualize accumulation in vivo (15, 16). However, fluorophores within close proximity (<4.5 nm) of Au are quenched and the fluorescence is diminished (17). This requires the growth of a spacer layer between the Au and fluorophore, making the overall NP size larger (13) or doping fluorophores within the nanoparticle itself, within a spacer layer (18). Fluorescence-based NP imaging also suffers from photobleaching and the loss of imaging function over time (19). An alternative imaging technique utilizes nanomaterials with attached magnetic resonance imaging (MRI) contrast agents to allow for noninvasive and high spatial and temporal resolution images (20, 21). The two main classes of MRI contrast agents are T_1_-weighted (longitudinal-bright contrast) and T_2_-weighted (transverse-dark contrast) contrast agents. T_1_-weighted MR images provide superior contrast and primarily consist of Gadolinium (Gd)-based chelates or Gd oxide nanomaterials and can be incorporated with NPs (22–26). While it is well understood that Gd(III) toxicity affects patients with nephrogenic systemic fibrosis (NSF) (27, 28), there is renewed concern of Gd deposition in the brain and bones of healthy individuals after Gd chelate ingestion (29, 30). We have previously demonstrated the incorporation of Gd-DOTA and Fe-DOTA within NMs to form Gd-NM (25) and Fe-NM (26) particles, respectively, in an effort to develop safer nanomaterials and reduce Gd exposure. While we observed a significant T_1_ MRI enhancement for both systems, the loading density of the chelate-based contrasting agents within the silica layer is limited due to the reduction of the relaxivity as Gd(III) and Fe(III) reach 2.5 × 10^5^ and 1.1 × 10^6^ contrast agents per NP, respectively. Furthermore, there is concern over the stability of these particles in the acidic tumor microenvironment, which is often more acidic than physiological pH, and can be as low as pH = 5.5 (31). For bifunctional NPs to be used for the combined imaging and treatment of tumors, they must be biologically stable in such an environment to maintain both function and safety.

Recently, Parchur et al. demonstrated effective imaging and treatment of colorectal cancer liver metastases using Gd_2_O_3_-coated nanorods (32). They reported a high relaxivity in terms of NP concentration (1.1 × 10^8^ mM^–1^ s^–1^), but did not address the stability of Gd(III) beyond scanning transmission electron microscopy (STEM) imaging. Furthermore, the lack of a protective outer Au shell for this particle geometry is likely to limit its physiological stability in the acidic tumor microenvironment. These findings suggest the development of an alternative bifunctional theranostic nanoparticle comprising an Au core, a Gd_2_O_3_ spacer layer, and an outer Au shell would overcome those potential limitations and address any Gd(III) stability concerns. Here, our primary goal was to develop a multilayered Au nanoparticle with a dense amount of internalized Gd(III) for bright MRI enhancement, and to evaluate the impact of pH on the stability of this new NP. We describe our synthesis of Gd_2_O_3_-NMs, measure the significant MRI contrast enhancement compared to previously reported chelate-based Gd-NMs, and demonstrate the stability of Gd within NMs under acidic conditions that mimic the tumor microenvironment.

## R**esults and discussion**

### Synthesis of Gd_2_O_3_-NM

**Fig. 1.**
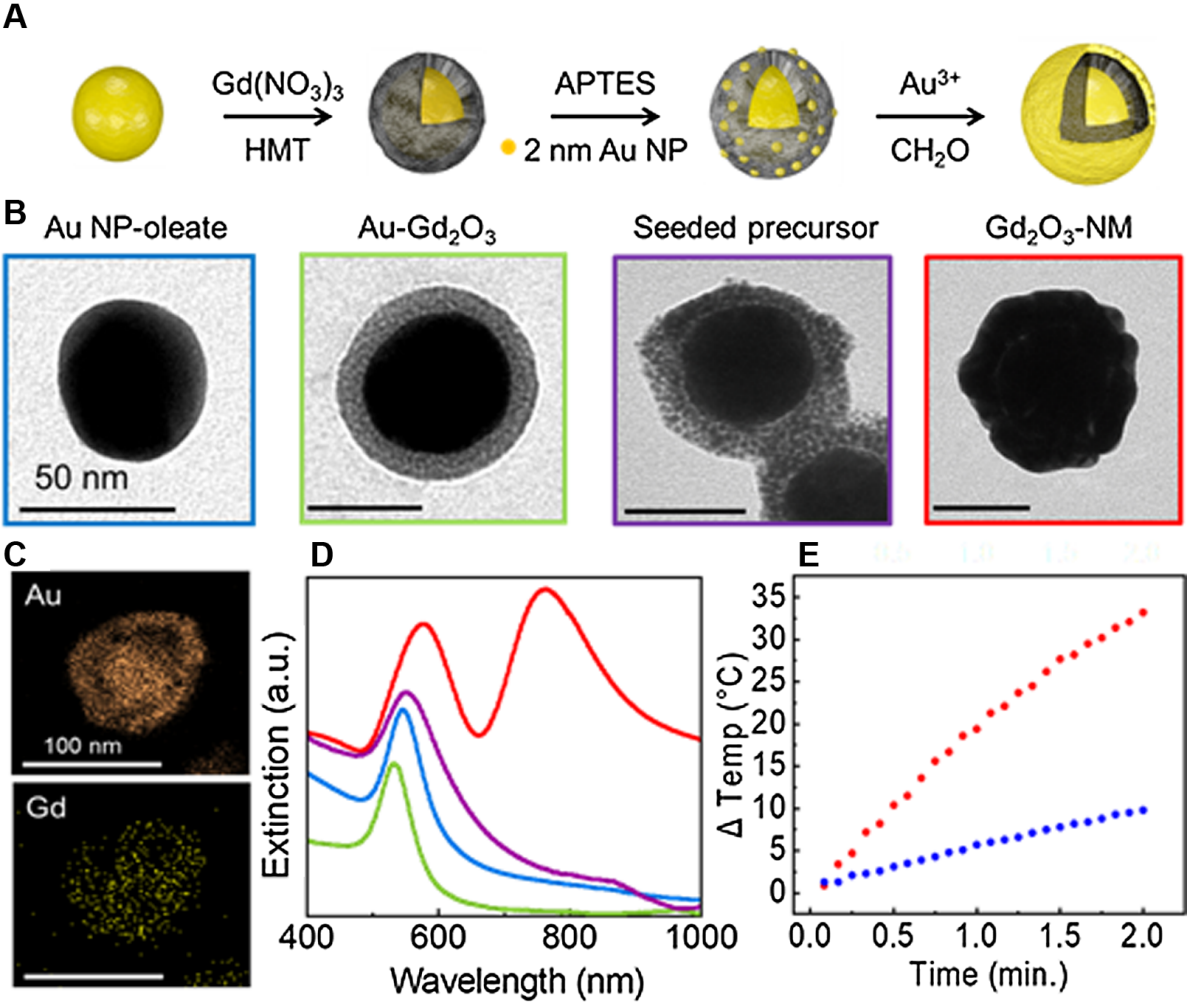
Gd_2_O_3_-NM synthesis and characterization. (A) Schematic diagram of the Gd_2_O_3_-NM synthesis showing the stepwise synthesis process: 50-m-diameter Au colloids coated with a Gd_2_O_3_ inner-shell, attached gold colloid to form the seeded precursor, and a continuous Au outer shell. (B) TEM images corresponding to each step in the process. (C) High-resolution STEM with high-angle annular dark field (STEM-HAADF) images showing elemental mapping of Au and Gd in the Gd_2_O_3_-NM. (D) extinction spectra of Au NP (blue), Au-Gd_2_O_3_ (green), seeded precursor (purple), and Gd_2_O_3_-NM (red). (E) photothermal heating of Gd_2_O_3_-NM (red) and water (blue) under near-IR irradiation (808-m-diode laser, 3 W cm^-2^, 2 min, 1 × 10^9^ NP mL^-1^).

A schematic representation of the Gd_2_O_3_-NM synthesis is shown in Fig. 1A. Gd_2_O_3_-NM synthesis was developed through substantial adaptations of the Au NR-Gd_2_O_3_ (32) and Gd-NM (25) protocols. The high interfacial energy between Au and Gd_2_O_3_ makes direct coating of Gd_2_O_3_ on Au nanoparticle cores quite challenging. One strategy introduces oleate as a compatible ligand for growing a Gd_2_O_3_ layer onto the Au core, to allow for tuning of the interfacial properties (Supplementary Material for synthesis specifics) (33). Initially, the surface of citrate capped Au NPs was modified with sodium oleate through surfactant exchange. The oleate appears to form a packed head-to-tail bilayer of around the Au NPs, where the carboxylic acid group of the first layer of the oleate binds to the surface of Au NPs. The second layer of oleate molecules intercalates into the first oleate layer via hydrophobic interactions, leaving its carboxylic acid group extending outward from the NP core surface. This bilayer serves as a bridge between the Au NP core and the initial layer of Gd^3+^ ions. A solution of Gd^3+^ ions (Gd(NO_3_)_3_) is added and adsorbed onto the oleate coated-Au NPs, most likely due to the carboxylic acid group of the outer oleate layer chelating the Gd^3+^ ions (34). The chelated Gd^3+^ ions serve as nucleation sites for growth of the Gd_2_O_3_ shell. Specifically, Gd(NO_3_)_3_ provides the Gd^3+^ ions and hexamethylene tetramine (HMT) is added to solution, which protonates, supplying OH- for oxide formation when the solution is heated, forming the Au/Gd_2_O_3_ NPs. The surface of the resulting Au/Gd_2_O_3_ NPs is further modified with 2–3 nm Au colloid prepared using the Duff synthesis (35) and attached to the Gd_2_O_3_ surface of the particle through (3-aminopropyl)triethoxysilane (APTES) coupling. Continuous electroless plating of the outer Au shell is achieved through reduction of Au(III) by formaldehyde, with the 2–3 nm Au colloid serving as nucleation sites (36). Representative transmission electron microscopy (TEM) images corresponding to each successive step are shown in Fig. 1B and Supplementary Material, Fig. S1.

The surface charge after each successive layer was grown was measured using Zeta potential measurements (Supplementary Material, Fig. S2). Furthermore, high-resolution STEM with high-angle annular dark field (STEM-HAADF) images with elemental mapping show the dispersion of the Gd within Gd_2_O_3_-NM (Fig. 1C). In addition, the outer shell was functionalized with mPEG-SH (10,000 MW) molecules to improve stability and reduce aggregation before additional characterization.

### Photothermal heating studies of Gd_2_O_3_-NM

NIR-active plasmonic nanomaterials are effective in converting resonant light to localized heating for remission of cancerous tumors. Representative extinction spectra at each successive step in the Gd_2_O_3_-NM synthesis are shown in Fig. 1D. Gd_2_O_3_-NM (red) exhibits NIR absorption, similar to previous NM systems due to the plasmonic coupling between the Au core and outer Au shell (25). The theoretical extinction of Gd_2_O_3_-NM was also determined using full-wave electromagnetic simulations (FDTD, Lumerical 2020) (Supplementary Material, Fig. S3). In an effort to confirm similar photothermal heating compared to Gd-NM, a solution of Gd_2_O_3_-NM (1 × 10^9^ NP mL^-1^, 1.66 pM) was irradiated for 2 min using a NIR laser (808 nm, 3 W cm-^2^). The solution reached a temperature over 55°C after 2 min of irradiation, providing sufficient heating required to initiate hyperthermia within tumor cells (Fig. 1E, red) (6). The H_2_O control solution only reached 32°C under the same conditions (Fig. 1E, blue). These results suggest Gd_2_O_3_-NM could be used to selectively heat tumor cells for PTT while minimizing damage to healthy tissue.

### Evaluation of MRI contrast using Gd_2_O_3_-NM

**Fig. 2.**
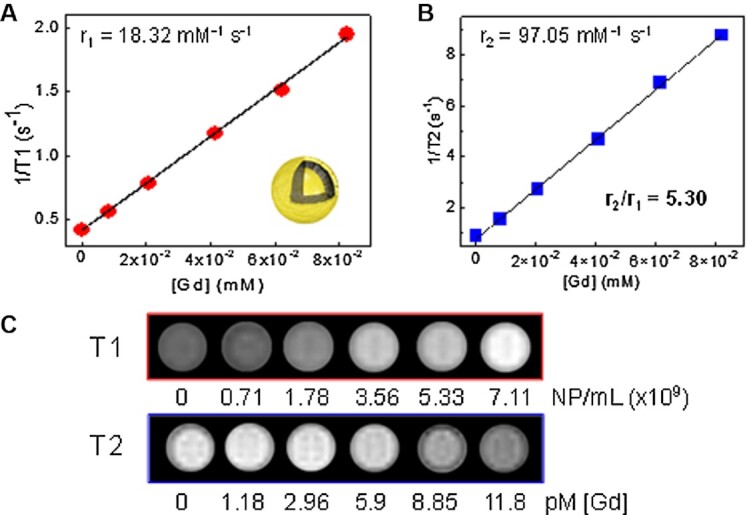
MRI properties of Gd_2_O_3_-NM. (A) r_1_ and (B) r_2_ relaxivity plots and (C) corresponding T_1_- and T_2_-weighted MRI images of Gd_2_O_3_-NM and concentration (NP mL^-1^ and pM [Gd]). The r_1_ and r_2_ values are determined by the slope of the linear plot of 1/T_1_ or 1/T_2_ versus [Gd] (mM^–1^ s^–1^).

We investigated the T_1_ and T_2_ MRI relaxivity enhancement of Gd_2_O_3_-NMs. The concentration of Gd(III) was evaluated using inductively coupled plasma mass spectrometry (ICP-MS). For the MRI measurements, the Gd(III) concentration was 8, 21, 41, 62, and 82 µM and measured at 4.7 T. The T_1_ relaxivity was determined by plotting the 1/T_1_ time (s^–1^) versus the Gd(III) concentration (mM). The MRI relaxivity (r_1_) as a function of Gd(III) concentration was 18.32 mM^–1^ s^–1^ (Fig. 2A), an enhancement of 1.8x compared to ultrasmall (1–2 nm) Gd_2_O_3_- alone (37). While the surface to volume ratio of Gd in Gd_2_O_3_-NM is less than ultrasmall Gd_2_O_3_, which would lower the T_1_ relaxivity, the decreased tumbling rate of the Gd centers in Gd_2_O_3_-NM is the likely cause of the observed enhancement of the T_1_ relaxivity (38). The outer Au shell also increases the distance between the Gd(III) centers and neighboring water molecules, resulting in a slight reduction of the r_1_. Before the addition of the Au shell, the measured r_1_ of the seeded precursor was 23.16 mM^–1^ s^–1^ (Supplementary Material, Fig. S4). However, the outer Au shell enables NIR absorption, provides a platform for additional functionalization, and improves biocompatibility. Furthermore, the larger shell of Gd in Gd_2_O_3_-NM results in an increase in the T_2_ relaxivity (97.05 mM^–1^ s^–1^, Fig. 2B). The simultaneous enhancement of both the r_1_ and r_2_ an r_2_/r_1_ ratio of 5.30 enables dual T_1_/T_2_ MR imaging strategies using Gd_2_O_3_-NM. The corresponding T_1_ and T_2_ images at each concentration show increasing brightness under T_1_ and darkening under T_2_ parameters (Fig. 2C). The dual T_1_/T_2_ MRI functionality of Gd_2_O_3_-NM could potentially be used to overcome a variety of imaging artifacts (39).

### Comparison of MRI contrast with Gd_2_O_3_-NM and Gd-NM

Our previous Gd-NM nanostructure utilized molecular chelates doped in a silica spacing layer between an Au core and an outer Au shell and exhibited a higher r_1_ relaxivity in terms of Gd(III) concentration (24 mM^–1^ s^–1^) (25). However, when comparing nanoparticle-based therapeutics that contain contrast agents, it is more relevant to consider the relaxivity in terms of particle concentration (32). Gd_2_O_3_-NM significantly improves upon chelate-doped Gd-NMs by increasing the number of Gd(III) per NP while maintaining a high T_1_ relaxivity. The previous design limited the increase of Gd(III) concentration within the NM, because the higher packing density of Gd(III) chelates caused the T_1_ relaxivity to drop significantly for concentrations above 2.5 × 10^5^ Gd(III) per NM (25).

**Fig. 3.**
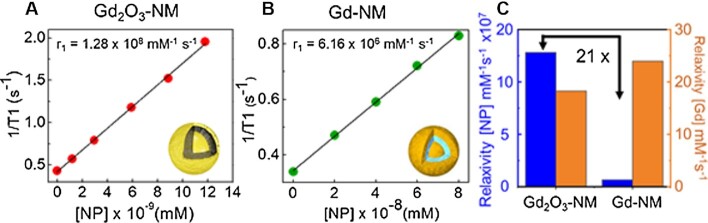
T_1_ MRI enhancement based on NP concentration. r_1_ relaxivity plots of (A) Gd_2_O_3_-NM and (B) Gd-NM. The r_1_ values are determined by the slope of the linear plot of 1/T_1_ versus [NP] (mM^–1^ s^–1^). (C) Comparative r_1_ relaxivity values for Gd_2_O_3_-NM and Gd-NM in terms of [NP] (blue) and [Gd] (orange).

Using the density of Au, we calculated that the mass of Au in a single Gd_2_O_3_-NM is 9.74 × 10^–5^ µg with dimensions [r_1_, r_2_, r_3_] = [25, 35, 50] nm. The Au and Gd content of Gd_2_O_3_-NM was measured using ICP-MS. The concentration of Gd(III) per NM in Gd_2_O_3_-NM was calculated as 6.98 × 10^6^ Gd(III) ions/NM, a 28× increase compared to Gd-NMs. Here, the T_1_ relaxivity data for Gd_2_O_3_-NM and Gd-NMs were calculated in terms of NP concentration and compared (Fig. 3A and B). Gd_2_O_3_-NM exhibits a 21× larger T_1_ relaxivity (1.28 × 10^8^ mM^–1^ s^–1^) in terms of NP concentration. This MRI enhancement in terms of particle concentration will enable the monitoring of NP accumulation during potential PTT applications.

Since less than 1% of NPs reach the tumor site after intravenous injection in the case of the EPR effect, the MRI enhancement per NP is a critical metric to estimate success in imaging applications (40). We previously demonstrated intravenously injected NM to passively target and treat triple negative breast cancer in mouse models (12). The concentration of NM in the tumor was 1.6 × 10^9^ NP mL^-1^, as determined by the biodistribution experiments. Based on the MRI data presented for Gd_2_O_3_-NM here, it is reasonable to suggest that sufficient MRI contrast would be observed at this Gd_2_O_3_-NM concentration, since it is within the range of concentrations shown. However, MRI data for our previous, chelate-based Gd-NM required a particle concentration of 1 × 10^10^ NP mL^-1^ for sufficient contrast, which in that case would require a substantially increased dosage to enable imaging contrast. For this reason, we believe Gd_2_O_3_-NMs are substantially more promising for future streamlined therapeutic strategies integrating MRI imaging and photothermal treatment.

### Gadolinium pH-dependent leaching in Gd_2_O_3_-NM versus Gd-NM

**Fig. 4.**
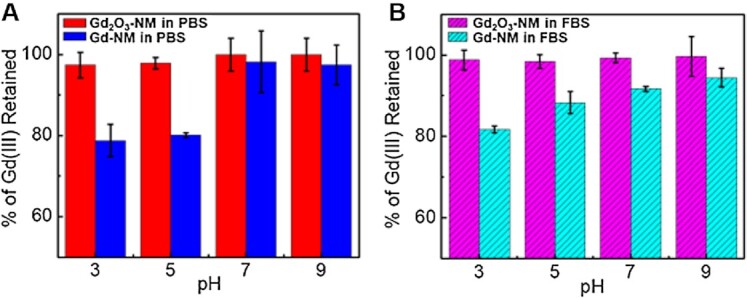
Stability of Gd(III) within NM. Percentage of Gd(III) retained within (A) Gd_2_O_3_-NM (red) and Gd-NM (blue) in PBS and (B) Gd_2_O_3_-NM (pink) and Gd-NM (aqua) in FBS after 5 days’ incubation, where pH = 3, 5, 7, and 9.

We investigated the relative stability of both Gd_2_O_3_-NM and chelate-based Gd-NM by measuring any potential Gd(III) leaching in various pH environments. The Gd(III) within Gd_2_O_3_-NM exists as an oxide, while the Gd(III) within Gd-NM is chelated and doped within the silica network. Both NPs have a protective outer Au shell to minimize leaching, improve NP stability, and provide a surface for additional functionalization, as needed. Solutions of Gd_2_O_3_-NM and Gd-NM were prepared (1 × 10^9^ NP mL^-1^, 1.66 pM) in phosphate-buffered saline (PBS) and fetal bovine serum (FBS) with the pH adjusted to 3, 5, 7, and 9. The solutions were incubated for 5 days (99°C), then centrifuged to collect the supernatant and measure Gd(III) using ICP-MS. The amount of Gd(III) measured from the supernatant and Gd(III) from a control sample were used to calculate and plot the % of Gd(III) retained within the NP. Gd_2_O_3_-NM exhibited great stability even in the most acidic environment (97% in PBS, Fig. 4A and 99% in FBS at pH 3, Fig. 4B). Although the chelate-based Gd-NM demonstrated 98% retention of Gd(III) in PBS at pH of 7, our results suggest less stability in more acidic environments. In PBS, Gd-NM retained only 79 and 80% of Gd(III) at pH of 3 and 5, respectively (Fig. 4A, blue). One possible explanation for the increased stability of Gd_2_O_3_-NM in acidic environments is due to the strong binding constant of Gd_2_O_3_; the dissociation constant of Gd_2_O_3_ is 1.8 × 10^−23^ (41, 42). The outer Au shell may also reduce etching and reshaping of the Gd_2_O_3_ layer. In contrast, the stability of Gd within the chelate-based Gd-NM requires the preservation of the molecular chelate itself in addition to the APTES coupled chelate in the silica (SiO_2_) network. It is possible that the acidic environment leads to the cleaving of those bonds and the leaching of Gd(III). At this time, it is not clear whether the leaching Gd(III) in Gd-NM is in the form of free Gd(III) or Gd chelate (Gd-DOTA). If it is the former, significant toxicity concerns would exist. If it is the latter, the toxicity concerns would be less due to the approved clinical use of Gd chelates, but the leaching of Gd chelates would reduce the MRI enhancement of Gd-NM. Meanwhile, we have demonstrated the stability of Gd_2_O_3_-NM in acidic environments and its superior MRI enhancement.

## Conclusions

We have demonstrated the synthesis of Gd_2_O_3_-NM and shown its effectiveness for NIR photothermal heating and MRI enhancement. We confirmed that significantly more Gd(III) can be loaded within Gd_2_O_3_-NM compared to our previous Gd-chelate-based Gd-NM system while maintaining an enhanced T_1_ relaxivity (r_1_) in terms of Gd concentration. This combination of high relaxivity and increased concentration of Gd(III) per NP in Gd_2_O_3_-NM resulted in a 21× enhancement of r_1_ in terms of NP concentration (1.28 × 10^8^ mM^–1^ s^–1^ versus 6.16 × 10^6^ mM^–1^ s^–1^ for Gd-NM). We have also demonstrated the dual T_1_/T_2_ MRI enhancement properties of Gd_2_O_3_-NM with an r_2_/r_1_ of 5.3 and shown viable MR imaging at concentrations below 2 × 10^9^ NP mL^-1^ and 20 µM [Gd]. We also investigated the stability of Gd(III) within Gd_2_O_3_-NM and found minimal leaching under acidic environments (<2% in FBS at pH of 3), while Gd(III) release from the chelate-based Gd-NM was as high as 18% under the same conditions. The Gd_2_O_3_-NM bio-system is a promising, non-invasive, and localized PTT option that could significantly reduce patient morbidity by selectively destroying tumor cells when illuminated while minimizing damage to healthy tissue. In addition, the T_1_-MRI functionality could enable clinicians to confirm Gd_2_O_3_-NM location and concentration prior to treatment, ultimately optimizing accumulation in tumor and increasing the effectiveness of the overall PTT procedure.

## Supplementary Material

pgac140_Supplemental_File

## Data Availability

All data needed to evaluate the conclusions of this study are included in the manuscript.
