# Supplementary material for: Plasmonic gadolinium oxide nanomatryoshkas: bifunctional magnetic resonance imaging enhancers for photothermal cancer therapy
Source: PNAS Nexus. 2022 Jul 29;1(4):pgac140. doi: 10.1093/pnasnexus/pgac140 (PMC9802487; doi:10.1093/pnasnexus/pgac140)
Supplement: pgac140_Supplemental_File [file pgac140_supplemental_file.pdf]

Supporting Information

Plasmonic Gadolinium Oxide Nanomatryoshkas: Bifunctional Magnetic Resonance Imaging Enhancers for Photothermal Cancer Therapy

Luke Henderson,[1,4] Oara Neumann,[2,4] Yara Kadria-Vili,[5] Burak Gerislioglu,[3,4] James Bankson,[5] Peter Nordlander,[2,3,4] and Naomi J. Halas[1,2,3,4]

[1]Department of Chemistry, [2]Department of Electrical and Computer Engineering, [3]Department of Physics and Astronomy, and [4]Laboratory for Nanophotonics, Rice University, 6100 Main St, Houston, Texas 77005, United States

[5]Department of Imaging Physics, The University of Texas M.D. Anderson Cancer Center, 1515 Holcombe Boulevard, TX 77030, United States

#Correspondence Email: halas@rice.edu

KEYWORDS: nanomatryoshka, MRI contrast agents, gadolinium oxide

Materials and Methods

Materials. Tetraethoxysilane (TEOS), (3-aminopropyl)triethoxysilane (APTES), tetrakis(hydoxymethyl) phosphonium chloride (THPC), $Gd(NO_3)\cdot 6H_2O$, $Mn(NO_3)_2 \cdot 4H_2O$, $Fe(NO_3)_3\cdot 9H_2O$, and chloroauric acid ($HAuCl_4\cdot 3H_2O$) were purchased from Sigma Aldrich. Potassium carbonate anhydrous (K_2CO_3) was purchased from Fisher, mPEG-Thiol (MW = 10000) from Laysan Bio, Inc. and NaOH from Fisher was used without purification. 50 nm gold colloids citrate NanoXact were purchased from NanoComposix. Aqua regia was used to clean all glassware and stir bars, followed by thorough rinsing with distilled water, ethanol, and Milli-Q water in the final step. Milli-Q water (18.2 MΩ.cm at 25 °C, Millipore) was used to prepare all solutions and reagents without further purification.

Au/Gd_2O_3/Au Nanomatryoshkas (NMs) synthesis. Au/Gd_2O_3/Au NMs were prepared by growing a shell of Gd_2O_3 around Au cores followed by growth of an outer Au shell. Briefly, citrate-capped Au spheres (50 nm) were dispersed in 0.02 M sodium oleate (NaOA) and heated at 80 ºC for 1 hr. The resultant Au-NaOA was centrifuged at 3000 G for 20 min and redispersed in water. For a standard reaction, 30 mL of Au-NaOA was added to 300 mL of Milli-Q water and vortexed. Then, 7.8 mL of hexamethylene tetramine (HMT) (0.1 M) and 12 mL of Gd(III)-nitrate (0.01 M) were added, vortexed for 1 min followed by sonication for 15 min. The reaction mixture was then heated at 80 ºC for 1 hr in a sealed container and left overnight. The Au/Gd_2O_3 solution was then centrifuged at 3000 K G for 20 min and resuspended in 15 mL of EtOH. 150 μL of 10 % APTES

(v/v in EtOH) was added and gently stirred for 12 hr at room temperature then dialyzed in EtOH for 12 hr. The Au/Gd_2O_3-APTES solution was concentrated in water and added to 40 mL Duff colloid that was prepared in advance (2 weeks)[1] and 750 μL of NaCl (1 M). The solution was sonicated for 35 min and left undisturbed for 24 hr. A continuous Au outer shell was grown and then redispersed in mPEG-thiol (10 k), following the previously reported protocol.[2]

The concentration of Gd(III) in the NMs was determined using inductively coupled plasma mass spectroscopy (ICP-MS). The measurements were performed in a PerkinElmer NexION 300. First, the NM samples were dispersed in concentrated aqua regia (HNO_3/HCl 1:3) solution overnight. The resulting solution was diluted by 500 times with a solution consisting of 2 % v/v HNO_3 solution and used for ICP-MS analysis. The Gd(III) concentration was determined using a calibration curve made with gadolinium ICP/DCP standard solutions (Sigma).

Theoretical extinction calculation of Gd_2O_3-NM. Full-wave electromagnetic simulations were performed using finite-difference time-domain (FDTD, Lumerical 2020) software. In all simulations, the size of the spatial grids in all dimensions was set to 1 nm and perfectly matched layers (PMLs) were used to cover the simulation region. For the considered nanostructures, the complex refractive indices for Au and Gd_2O_3 were taken from Johnson[3] and Sahoo[4], respectively. The Courant stability factor (~0.99) for the simulated model was realized by setting the simulation time step to dt = 0.02 fs.[5] The extinction spectra were extracted using total-field scattered-field approach over the wavelength range of interest with a regular plane-wave with a pulse length of 75 fs.

Photothermal heating of Gd_2O_3-NM. A solution of Gd_2O_3-NM in H_2O was prepared (1×10^9 NP/mL) in an insulated glass beaker equipped with a stir bar and thermocouple. A second thermocouple was used to measure the ambient temperature. An 808 nm diode laser was set to 3

W/cm^2 and positioned above the stirring solution. The solution was irradiated for 2 min and the temperature of the solution and ambient temperature was recorded. As a control, the beaker was then filled with water and irradiated under the same conditions.

Stability of Gd_2O_3-NM and Gd-NM in various pH environments. Solutions of phosphate buffered saline (PBS) and fetal bovine serum (FBS) were pH adjusted to 3, 5, 7, or 9 by adding 1 M HCl or 1 M NaOH. 30 μL of Gd_2O_3-NM or Gd-NM (5 x 10^9 NP/mL) was added to 1 mL of each solution and vortexed for 1 min. Each sample was left in an incubator at 99 °Fs. After 5 days, the samples were centrifuged, and the supernatant was collected and dissolved in aqua regia. A control sample of 30 μL of Gd_2O_3-NM or Gd-NM (5 x 10^9 NP/mL) was also dissolved in aqua regia. The Gd(III) concentration was quantified using ICP-MS. The percent of Gd(III) retained within NM was calculated and reported.

Magnetic resonance characterization of Gd_2O_3-NM. All relaxation measurements were performed on a 4.7 T Biospec system (Bruker Biospin MRI) with a 30 cm bore, using imaging gradients with an inner diameter of 60 mm and a volume resonator with 35 mm inner diameter. Dilutions of the particle solution were sealed in 200 μL PCR tubes and placed in a holder. Spin-lattice (T1) relaxation times were measured using a RARE variable repetition time (TR) sequence [echo time (TE) = 9.9 ms; with 10 TRs from 15,000 to 400 ms]. Spin-spin (T2) relaxation times were measured using a multiecho sequence (TEmin = 172.7 ms, with 10-ms echo spacing over 30 echoes; TR = 1750 ms). All images were acquired with matching slice geometry (1 mm axial sections, 32 mm × 32 mm field-of-view over a 256 × 256 image matrix). Relaxation time constants for each sample were measured by fitting signal decay curves to a standard model in ParaVision 5, the operating software for the Biospec platform.

Instrumentation

Transmission electron microscopy (TEM) was performed using a JEOL 1230 operating at 80 kV. High-resolution STEM with high-angle annular dark field (STEM-HAADF) and and energy-dispersive X-ray element mapping were used for elemental analysis. Cary 5000 UV/Vis/NIR Varian spectrophotometer was used to measure the extinction spectra of the nanoparticles. DLS (Dynamic Light Scattering) and zeta potential (Malvern Zetasizer) measurements were performed to determine the particles' size, polydispersity index, and zeta potential. Inductively Coupled Plasma Mass Spectroscopy (ICP-MS) was used to determine the concentrations of Gd, Au and Si ions. Magnetic Resonance Imaging (MRI) at 4.7T was used to obtain the spin-lattice relaxation (T_1), spin-spin relaxation (T_2), T_1-, and T_2-weighted MR images at time constants for aqueous Gd_2O_3-NMs. A Diomed laser (CW-GaAlAs, 808 nm ± 20 nm) was used for PTT experiments.

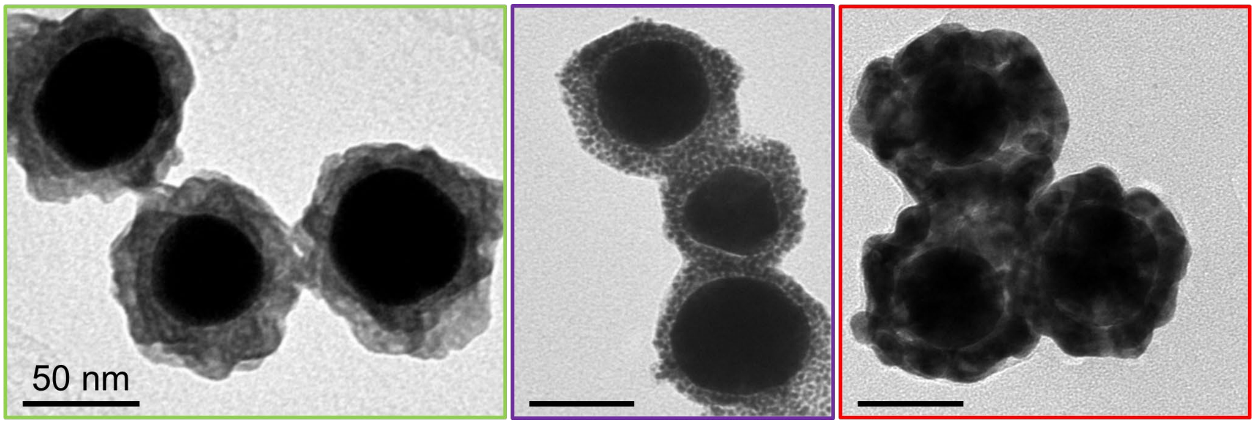

Figure S1. TEM images of: (i) Gd_2O_3-Au NP, (ii) 2nm Au NP-Gd_2O_3-Au NP, and (iii) Gd_2O_3-NM.

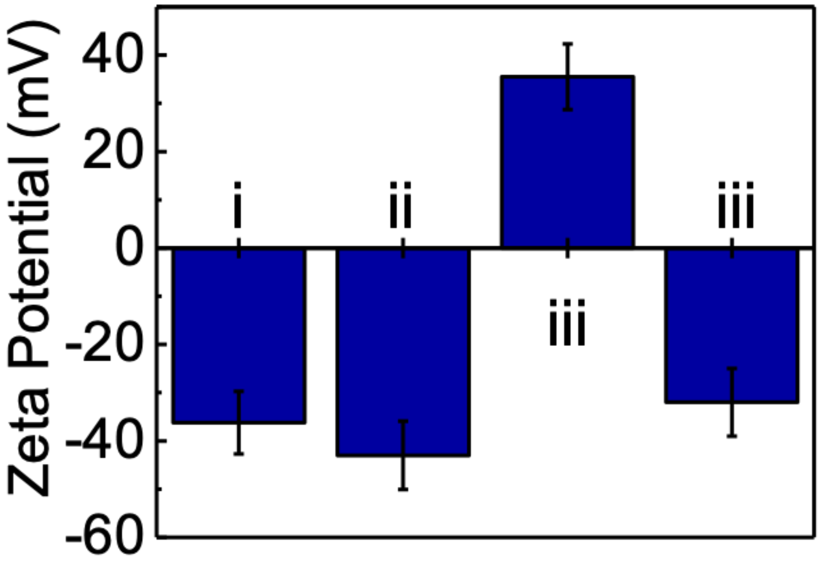

Figure S2. Gd_2O_3-NM surface characterization. Zeta potential measurements at synthetic steps: (i) Au NP-citrate, (ii) Au NP-NaOA, (iii) Gd_2O_3-Au NP, and (iv) Gd_2O_3-NM.

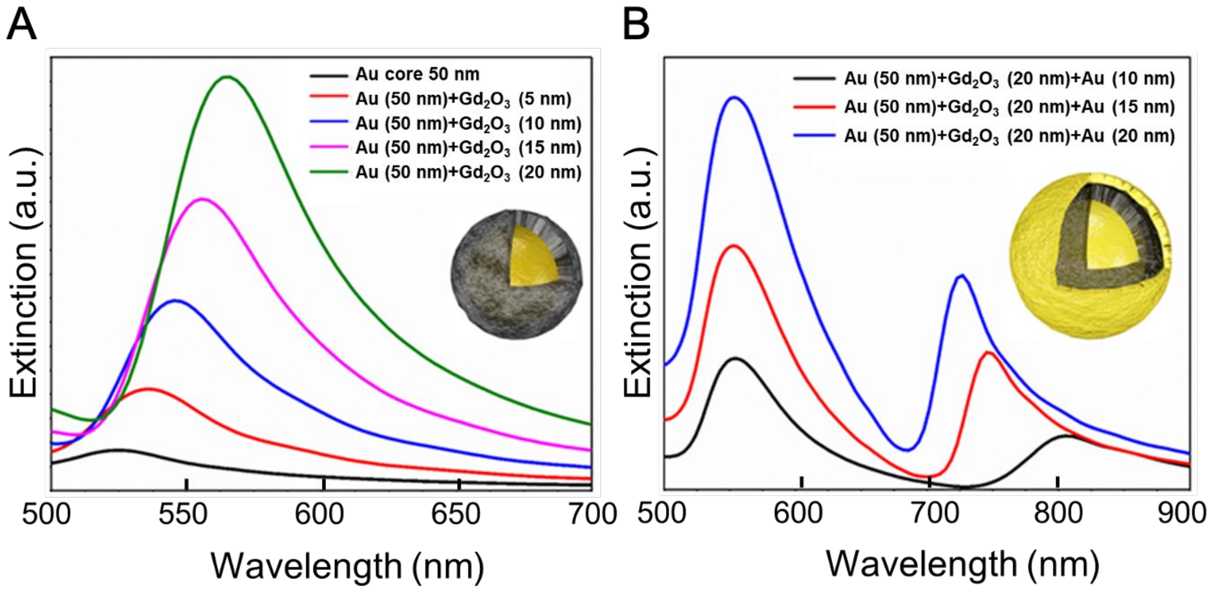

Figure S3. Theoretical extinction spectra. Calculated extinction spectra for (*A*) 50 nm Au nanosphere coated with varied Gd_2O_3 shell thickness and (*B*) 50 nm Au and 20 nm Gd_2O_3 NP with varied Au outer shell thickness, indicating a clear Fano effect.

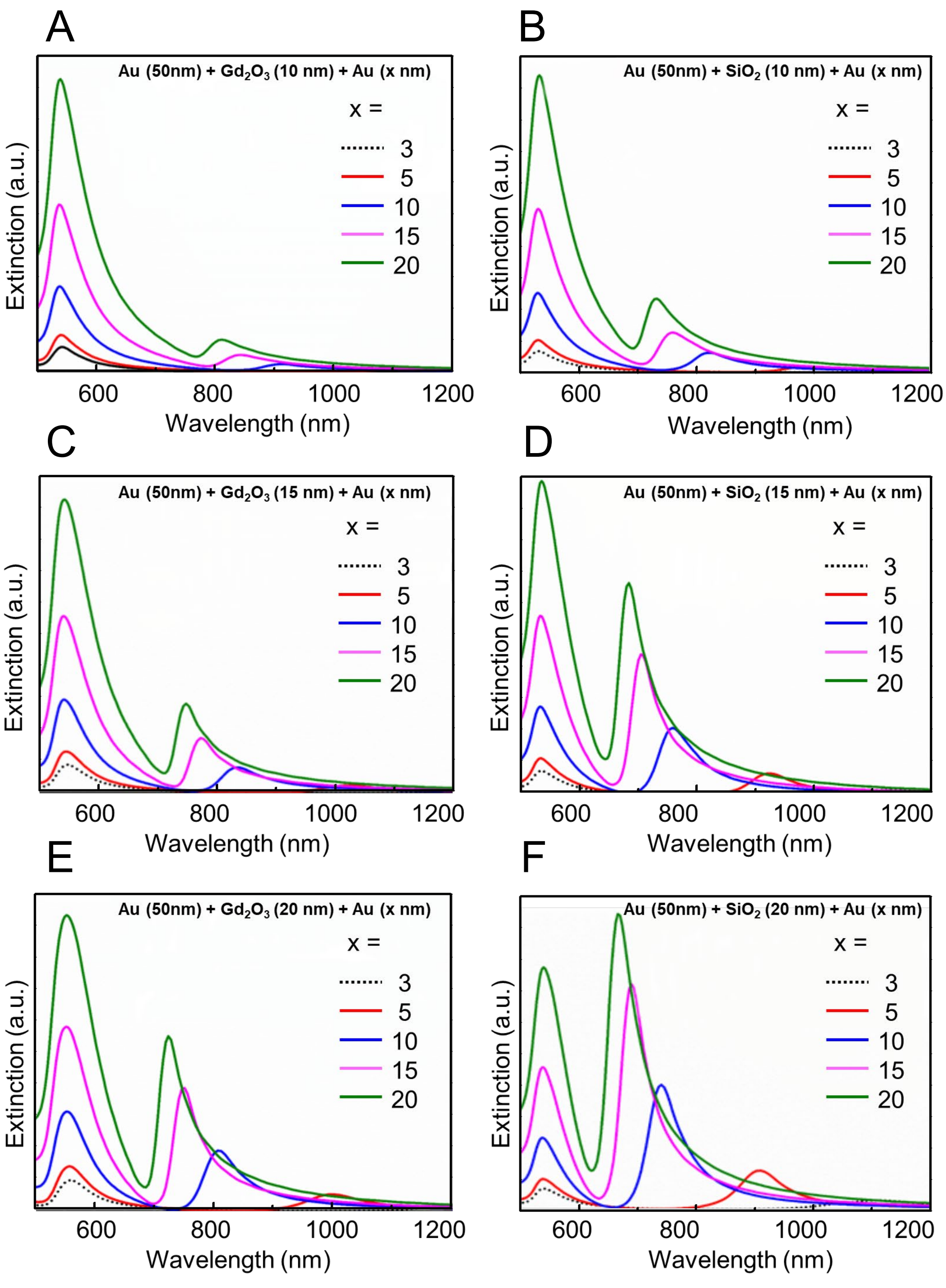

Figure S4. Theoretical extinction spectra. Calculated extinction spectra for Gd_2O_3-NM with 50 nm Au core coated with varied Gd_2O_3 shell thickness of: (*A*) 10 nm, *(B)* 15 nm, and *(C)* 20 nm and varied outer Au shell thickness of: (black) 3 nm (red) 5 nm, (blue) 10 nm, (magenta) 15 nm, and (green) 20 nm, indicating a clear Fano effect. Calculated extinction spectra for Gd-NM with 50 nm Au core coated with varied SiO_2 shell thickness of: (*D*) 10 nm, *(E)* 15 nm, and *(F)* 20 nm

and varied outer Au shell thickness of: (black) 3 nm (red) 5 nm, (blue) 10 nm, (magenta) 15 nm, and (green) 20 nm, indicating a clear Fano effect.

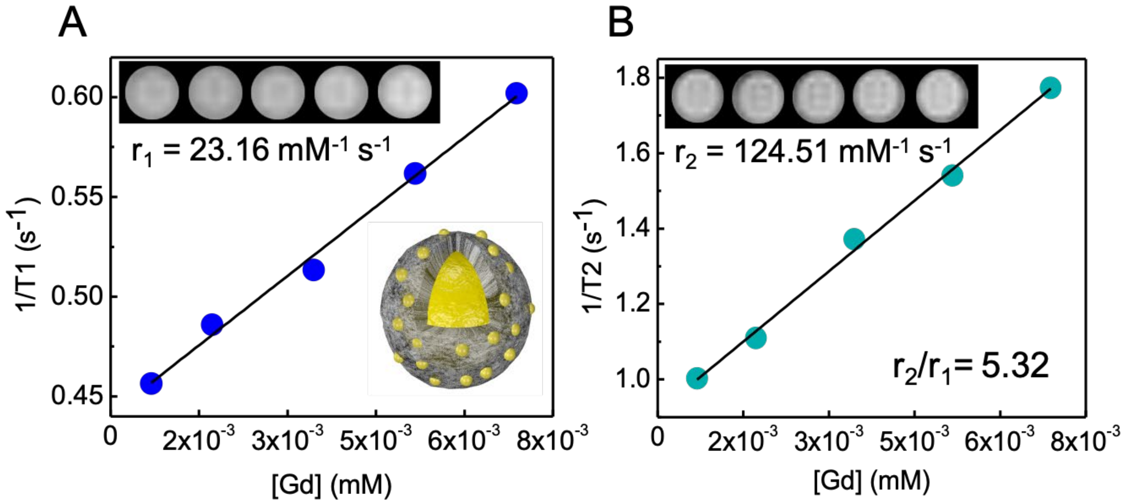

Figure S5. T1 and T2 relaxivity of seeded precursor. MRI relaxivity plots and images of seeded precursor using (*A*) T1 and (*B*) T2 parameters.

References

1. Duff, D. G.; Baiker, A.; Edwards, P. P., A new hydrosol of gold clusters. 1. Formation and particle size variation. *Langmuir* **1993,** *9* (9), 2301–2309.

2. Henderson, L.; Neumann, O.; Kaffes, C.; Zhang, R.; Marangoni, V.; Ravoori, M. K.; Kundra, V.; Bankson, J.; Nordlander, P.; Halas, N. J., Routes to Potentially Safer T1 Magnetic Resonance Imaging Contrast in a Compact Plasmonic Nanoparticle with Enhanced Fluorescence. *ACS Nano* **2018,** *12* (8), 8214-8223.

3. Johnson, J. B.; Christy, R. W. Optical constants of the noble metals. *Phys. Rev. B* **1972,** *6* (12), 4370-4379.

4. Sahoo, N. K.; Thakur, S.; Tokas, R. B. Growth-dependent refractive index nonlinearity and mean microstructural properties of codeposited composite gadolina silica films. *Appl. Opt.* **2006,** *45*, 3243-3252.

5. Zheng, F.; Chen, Z. Numerical dispersion analysis of the unconditional stable 3-D ADI-FDTD method. *IEEE Trans. Microwave Theory Tech.* **2001,** *49* (5), 1006-1009.
